# Multi-omic modeling of antidepressant response implicates dynamic immune and inflammatory changes in individuals who respond to treatment

**DOI:** 10.1371/journal.pone.0285123

**Published:** 2023-05-15

**Authors:** Shih-Chieh Fuh, Laura M. Fiori, Gustavo Turecki, Corina Nagy, Yue Li

**Affiliations:** 1 School of Computer Science, McGill University, Rue University, Montréal, Quebec, Canada; 2 Department of Psychiatry, McGill Group for Suicide Studies, Douglas Mental Health University, Montreal, Quebec, Canada; Brigham and Women’s Hospital and Harvard Medical, Channing Division of Network Medicine, UNITED STATES

## Abstract

**Background:**

Major depressive disorder (MDD) is a leading cause of disability worldwide, and is commonly treated with antidepressant drugs (AD). Although effective, many patients fail to respond to AD treatment, and accordingly identifying factors that can predict AD response would greatly improve treatment outcomes. In this study, we developed a machine learning tool to integrate multi-omic datasets (gene expression, DNA methylation, and genotyping) to identify biomarker profiles associated with AD response in a cohort of individuals with MDD.

**Materials and methods:**

Individuals with MDD (N = 111) were treated for 8 weeks with antidepressants and were separated into responders and non-responders based on the Montgomery–Åsberg Depression Rating Scale (MADRS). Using peripheral blood samples, we performed RNA-sequencing, assessed DNA methylation using the Illumina EPIC array, and performed genotyping using the Illumina PsychArray. To address this rich multi-omic dataset with high dimensional features, we developed integrative Geneset-Embedded non-negative Matrix factorization (iGEM), a non-negative matrix factorization (NMF) based model, supplemented with auxiliary information regarding gene sets and gene-methylation relationships. In particular, we factorize the subjects by features (i.e., gene expression or DNA methylation) into subjects-by-factors and factors-by-features. We define the factors as the meta-phenotypes as they represent integrated composite scores of the molecular measurements for each subject.

**Results:**

Using our model, we identified a number of meta-phenotypes which were related to AD response. By integrating geneset information into the model, we were able to relate these meta-phenotypes to biological processes, including a meta-phenotype related to immune and inflammatory functions as well as other genes related to depression or AD response. The meta-phenotype identified several genes including immune interleukin 1 receptor like 1 (IL1RL1) and interleukin 5 receptor (IL5) subunit alpha (IL5RA), AKT/PIK3 pathway related phosphoinositide-3-kinase regulatory subunit 6 (PIK3R6), and sphingomyelin phosphodiesterase 3 (SMPD3), which has been identified as a target of AD treatment.

**Conclusions:**

The derived meta-phenotypes and associated biological functions represent both biomarkers to predict response, as well as potential new treatment targets. Our method is applicable to other diseases with multi-omic data, and the software is open source and available on Github (https://github.com/li-lab-mcgill/iGEM).

## Introduction

Major depressive disorder (MDD) is a chronic and debilitating illness that affects 300 million people worldwide [1]. Its core symptoms are depressed mood and lack of interest, altered appetite and sleep, difficulty concentrating, and suicidal thoughts [2]. Between one- to two-thirds of MDD patients do not respond to their first line of antidepressant (AD) treatment, and up to one-third of patients fail to respond after multiple courses of AD treatment [3]. As such, identifying factors which can predict AD response could greatly improve treatment outcomes.

A variety of clinical and sociodemographic variables have been investigated as predictors of response, such as diagnostic symptomology, age, socioeconomic variables, and BMI [4, 5]. However, the use of molecular measures to predict response has become increasingly important, in part as they have not only the potential to predict response, but also to reveal underlying biological mechanisms [6–9]. Although the brain represents the most relevant tissue for identifying biomarkers of AD response, due to its lack of accessibility, the majority of studies rely on the investigation of peripheral markers [10]. A number of high-throughput studies have been performed in the blood, including those investigating gene expression, DNA methylation, genetic variation, and metabolic profiling, and have identified a number of factors related to AD response [11–17]. While promising, many of these findings have yet to be replicated, nor is it clear how findings from these different studies may be related. Moreover, it has become abundantly clear from genome-wide analyses that both MDD and AD response is highly polygenic and pleiotropic, each with small effect sizes. This creates challenges when using genome-wide approaches, particularly with smaller sample sizes, which may lack sufficient power to detect relevant, but small, differences. Additionally, it is likely that the molecular factors that predict AD response involve both static (ie, genetic variation) and dynamic effects (i.e., gene expression). Accordingly, the integration of multi-omic datasets may provide a greater capacity to predict response. While previous studies have implemented multi-omic approaches to study AD response and MDD, for example, by combining genotype and metabolite data [18], our study increases the dimensions by including three levels of molecular profiling and clinical data.

In the present study, we integrated gene expression, DNA methylation, genotype variation, and clinical questionnaire scores from a cohort of individuals with MDD who were treated with AD for 8 weeks, allowing us to generate a composite biomarker of AD response. To investigate this multi-omic dataset, we applied non-negative matrix factorization (NMF). Originally developed by Lee et. al. [19], NMF has been applied to the field of bioinformatics in various contexts, including cancer classification and feature clustering [20, 21]. It is a powerful framework for analysis of multi-omic datasets because it can distill latent factors from the data, which we often refer to as, *topics*, below. In this context, NMF can produce patient loading scores and feature basis scores, representing the relative importance or contribution of the patients and features (such as the genes) with respect to each topic. As a result, we can treat each topic as a *metagene* and perform hypothesis testing over these *metagenes*, which are much fewer than the total number of genes, thereby reducing the number of statistical tests being performed.

These characteristics of NMF make it suitable for multi-omic dataset analyses, and several researchers have applied NMF towards investigating MDD, with promising results. Shao et. al. used a cluster-driver NMF to discover common and distinct brain structural connectivity patterns between individuals with schizophrenia and MDD [21]. In addition, a recent pilot study by Chen et. al. applied NMF on the 30-item Inventory of Depressive Symptomatology-Clinician Rating (IDS-C30) to derive subtypes of treatment-resistant depression (TRD) and identified biomarkers with differential expression between the subtypes, including several immune and inflammation regulators such as *IL-1β*, *IL-6*, and *TNF-α* [22]. However, NMF alone does not directly reveal pathway or geneset information and often requires *post-hoc* geneset enrichment analysis to interpret the topics.

In this study, we present integrative Geneset-Embedded non-negative Matrix factorization (iGEM) to computationally address the above-mentioned challenges. Briefly, iGEM is a joint NMF model with sparsity constraint and biological network regularization. To guide topic learning, iGEM exploits known geneset information by further factorizing the feature factor score matrix into topics-by-genesets and genesets-by-genes. In this study, we applied iGEM to multi-omic data to derive shared factor scores of genes, genesets, and methylation sites for the relative importance in each topic. With the factor scores, we performed additional analyses to incorporate genotype and clinical feature information, to better understand the relationships between molecular processes and MDD, and how they relate to AD response.

## Results

### Analysis overview

**Fig 1:**
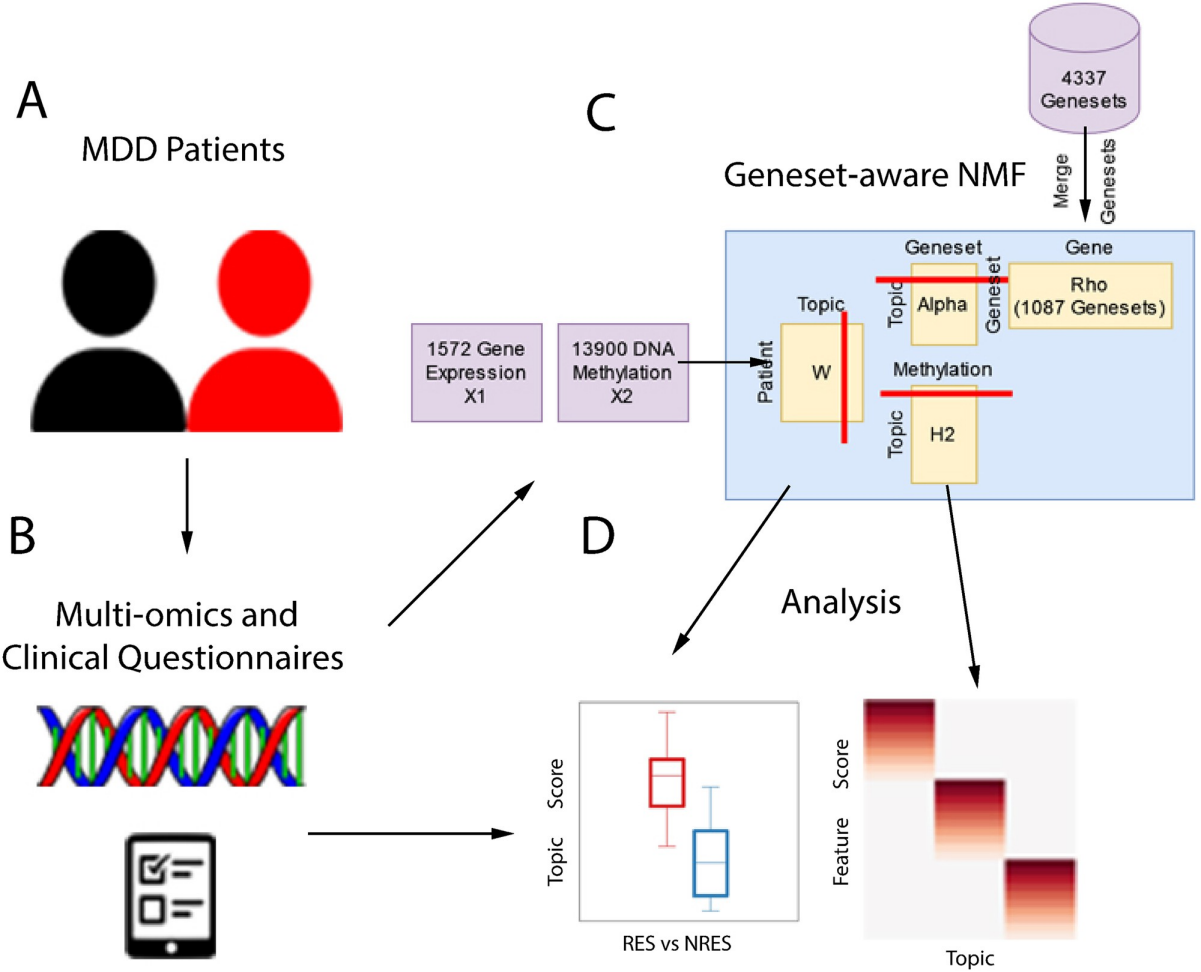
Overview of the study design. (A) We collected blood samples and questionnaire responses from 111 MDD subjects, among whom 61 were responders (RES) and 50 were non-responders (NRES) following 8 weeks of antidepressant treatment with an SSRI or SNRI. (B) The collected samples from these subjects before antidepressant treatment were profiled with gene expression (X1) by RNA-seq and DNA methylation (X2) by the methylationEPIC array. (C) The gene expression and DNA methylation were provided as input to our iGEM, a geneset-aware NMF approach. iGEM factorizes them into several matrices, including patient meta-phenotype scores (W), genomic feature factor score for gene (H1) and methylation (H2), where H1 can be further decomposed into geneset factor score (Alpha) and known gene-geneset information (Rho). (D) We associate the patient meta-phenotype scores with their response to antidepressant treatment and then identify corresponding top genomic features.

Our approach to identifying factors predicting AD treatment response is shown in Fig 1. Our patient cohort consisted of 111 individuals with MDD who were treated with AD for 8 weeks, after which they were classified as responders (RES, N = 61) or non-responders (NRES, N = 50) based on change in Montgomery–Åsberg Depression Rating Scale (MADRS) scores between baseline and week 8. Using peripheral blood samples, we collected three high-throughput molecular datasets: RNA-sequencing, DNA methylation, and SNP genotyping. We first identified gene expression and DNA methylation differences related to response, which were used as input for our iGEM model. The model identified several sets of AD-related factors, which were investigated in downstream analyses, including their relationship to depressive symptoms, and genetic variation.

### Differential analyses

**Fig 2:**
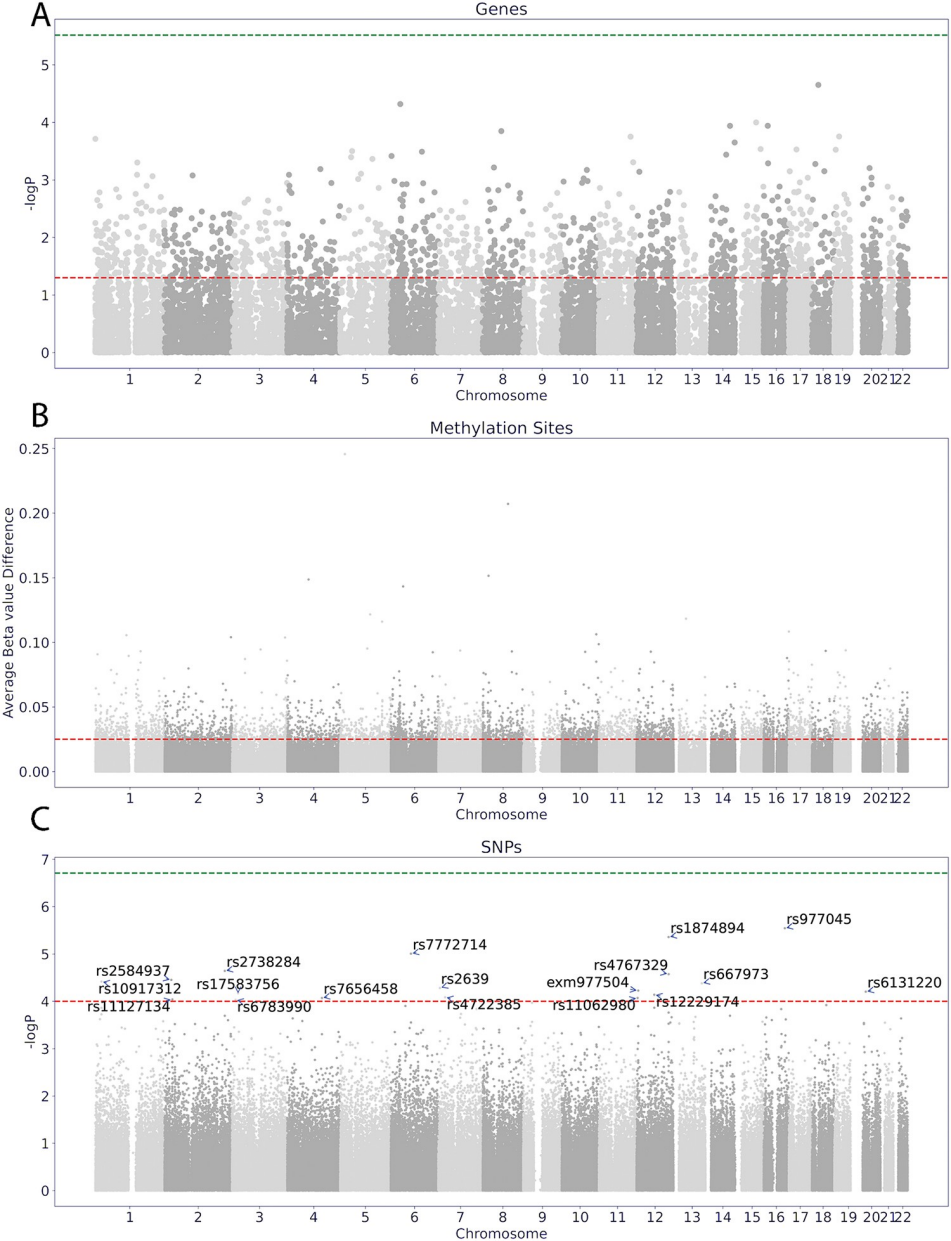
Manhattan plots for differential analysis. (A) Manhattan Plot for differential analysis of gene expression. (B) Manhattan Plot for differential analysis of methylation sites. (C) Manhattan Plot for differential analysis of SNPs. The green horizontal line indicates the negative log P-value where P = 0.05 / N (N = 16570, 673038, and 258782 for genes, methylation sites, and SNPs respectively). The red line indicates the differential features taken as inputs by the model (gene expression and methylation) or for further analysis (SNPs).

We first performed differential analysis for the gene expression and DNA methylation datasets, focusing on autosomal chromosomes and protein coding genes, to identify differences related to AD response. The results are shown in Fig 2. Not surprisingly, none of the features passed the stringent Bonferroni-corrected FDR of 0.05 in Fig 2, partly due to the small sample size of our cohort. Nonetheless, we opted to further explore the data from a multi-omic perspective using our iGEM model by setting a lenient threshold to select as many associated candidate markers as possible. We selected genes and methylation sites that showed nominally significant differences between RES and NRES, at the p-value less than or equal to 0.05 and absolute average beta value difference greater than or equal to 0.025, for gene expression and methylation, respectively. Genesets based on the differential genes were selected from the Bader genesets described in the Methods section. A total of 1572 genes, 13900 methylation sites, 283740 SNPs, and 1087 genesets were used for our model, and downstream analyses.

### Relationship of AD response with multi-omic latent factors

**Fig 3:**
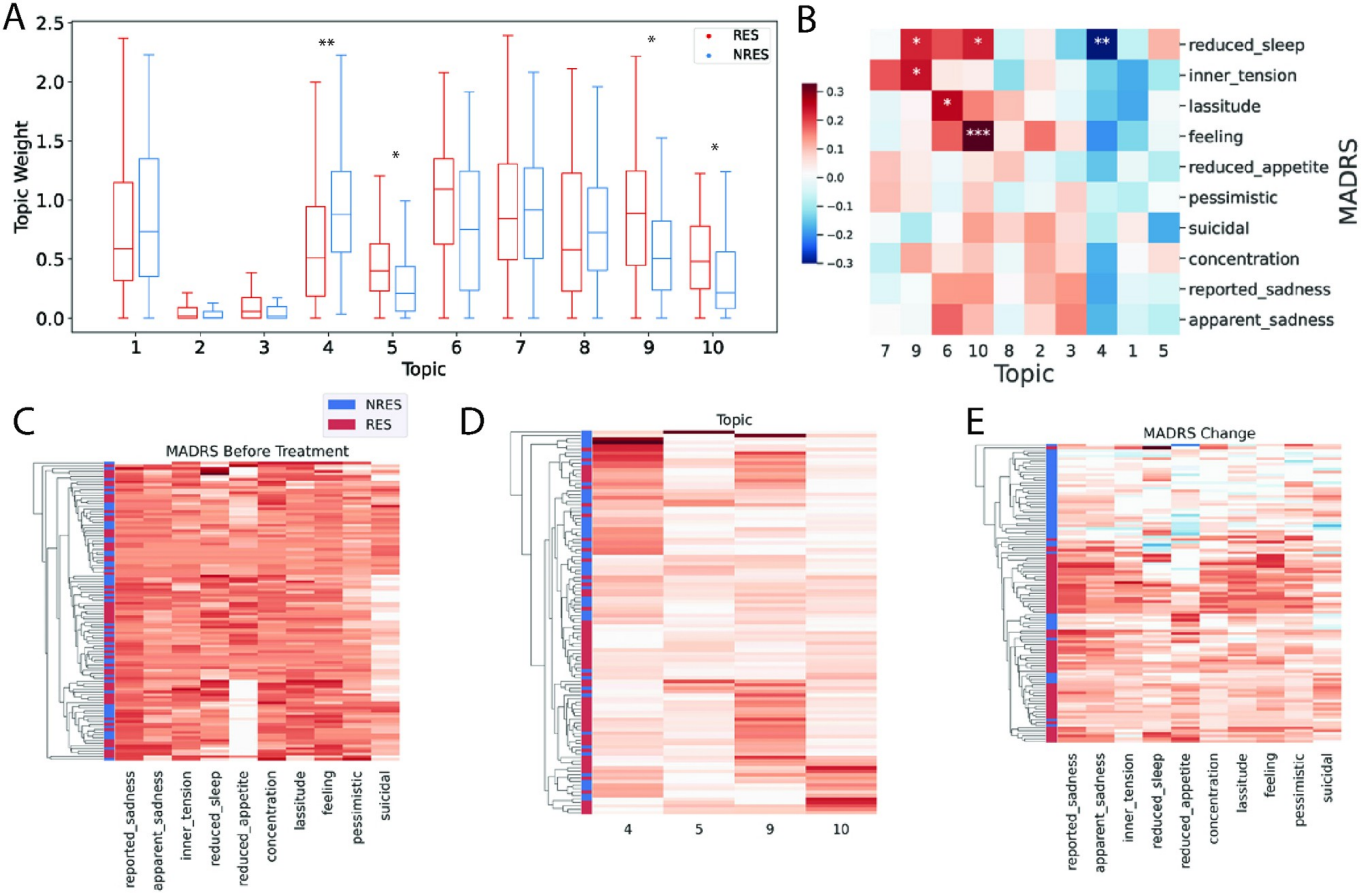
(**A**) Boxplot for patient factor score distribution between RES and NRES. The box indicates the range between the upper and the lower quantile. The line shows the median value. The whiskers indicate the last point within 1.5 interquantile range. *, ** represents corrected P-value < 0.05, 0.001 respectively, based on spearman correlation. (**B**) Pearson correlation between the patient factor score and the MADRS response following treatment. The color bar shows the magnitude and sign of correlation. Topics are arranged by columns, and the MADRS responses are arranged by rows. *, **, *** represents corrected P-value < 0.01, 0.05, and 0.001 respectively. (**C**) Hierarchical clustering of patients based on baseline MADRS score. The color intensity is proportional to the scores. (**D**) Hierarchical clustering of patients based on patient meta-phenotype scores of the top 4 topics. The topic scores were normalized within the range of 0 and 1. (**E**) Hierarchical clustering of patients based on the change in MADRS scores. The change in MADRS response is defined as a ratio of (week 0 score–week 8 score) / week 0 score. A greater ratio represents a greater improvement. The red and blue color intensities are proportional to the decrease and increase of MADRS scores, respectively. We define RES as (week 0 score–week 8 score) / week 0 score ≥ 0.5 and NRES as (week 0 score–week 8 score) / week 0 score < 0.5 respectively.

We applied iGEM to jointly model the filtered gene expression and methylation features. We used the Bader genesets to guide the learning of the latent factors. We performed a number of trials varying the latent factor number, and determined that 10 latent factors best captured the multi-omic dataset. We represented each subject by 10 disease factor scores, referred to as “topics”. To interpret the clinical meaning of these 10 topics, we correlated them with the AD treatment response status. We identified four significantly-correlated topics (BH-corrected FDR < 0.05). In particular, topics 5, 9, and 10 have higher factor scores in RES, while topic 4 has a higher factor score in NRES (Fig 3A, **S2 Table in** S2 File). This suggests that the top gene or methylation features in topics 5,9 and 10 are associated with AD response. On the other hand, the top genes and pathways in topic 4 may be related to the AD resistance of NRES patients.

We next examined the pattern of patient factor scores with respect to AD response by ordering the patients on topic 10 because due to its clinical significance and biology (described next). As shown in Fig 3A, RES patients in topic 10 have a higher score than NRES patients. This is consistent with the results presented in Fig 3B, where the topic 10 factor score was positively correlated with all MADRS responses.

To investigate the relationship between AD-related topics and depressive symptoms, we correlated the topics with the 10 individual MADRS items. The 10 MADRS items include “reported sadness”, “apparent sadness”, “pessimistic”, “suicidal”, “concentration”, “lassitude”, “feeling”, “inner tension”, “reduced sleep”, and “reduced appetite”. We created scores for each MADRS item by subtracting values post-AD treatment from baseline values then dividing by the baseline value, such that response to AD can be expressed by a decrease of each item, which represents a recovery from the depressed state. The correlation analysis between each MADRS item and the patient factor score was performed, and the significantly correlated pairs are shown in **S3 Table in** S2 File. Five pairs were found to be significant after correction (BH-corrected FDR < 0.05). Three topics were related to “reduced sleep”: topics 4, 9, and 10. Additionally, topic 10 was related to “feeling”, and topic 9 was related to “inner tension”. Furthermore, we have also performed hierarchical clustering for the patients based on MADRS score change, topic scores from the four AD-related topics, and MADRS score before treatment. We observed that the baseline MADRS scores cluster responder (RES) and non-responder (NRES) patients poorly (Fig 3C). In contrast, the top 4 meta-phenotypes, namely topics 4, 5, 9, and 10 selected based on the Spearman correlation p-values with the RES and NRES led to much better separation of RES and NRES (Fig 3D). However, note that while the patient meta-phenotype scores were derived from gene expression and DNA methylation of the patients before treatment, these were top features chosen by the differential analysis comparing RES and NRES. Therefore, the results do not completely reflect the generalization of these topics on the unseen patient data. The clustering result based on all topics are shown in **S2 Fig in** S2 File, and we have highlighted a group of patients with higher score in topic 10, which is consisted of mostly RES patients. As a reference, we also performed the clustering using the change of the 10 MADRS items after treatment, which serves as an upper bound of the clustering quality as the response labels were derived based on those 10 MADRS item scores (Fig 3E).

### Genes and genesets related to AD-response

**Fig 4:**
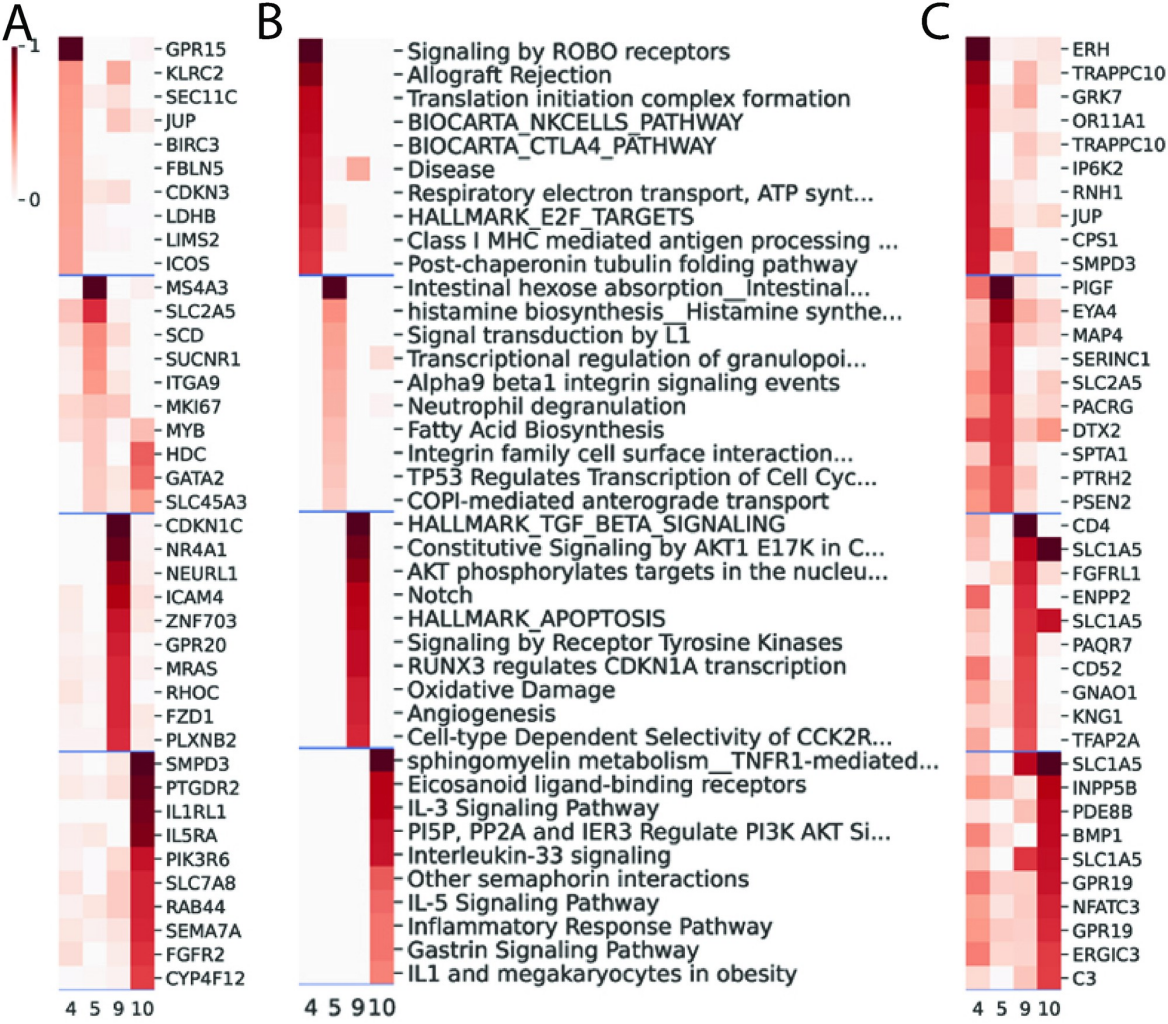
Top 10 features by feature factor score. (A) Top 10 features by feature factor score of gene. (B) Top 10 features by feature factor score of geneset. (C) Top 10 features by feature factor score of the most correlated proximal gene of methylation sites. The factor scores of the features are arranged in decreasing order of rank from top to bottom. The top 10 rows are from the top 10 features in the first topic. The next 10 rows are the top features from the next topic, and so on. For methylation, the names of the top methylation sites were substituted with its most correlated gene among proximal genes within 500kb, determined by Pearson correlation. The color represents the relative score of a feature in each topic within a range of 0–1. The top feature in each topic has a score of 1, and the score of other features are presented as a proportion of the top feature score within the same topic.

We examined the top genes, genesets, and DNA methylation-related genes for each of the four AD-related topics (Fig 4). The top features were selected based on the feature factor score within each topic. The top geneset of topic 4 is related to the ROBO pathway, and studies have shown that upregulation of ROBO signaling is related to depression in both humans and mice [23]. On the other hand, suppression of ROBO signaling has an antidepression effect, which is mediated by an increase of neuronal cytoskeleton remodeling and neuroplasticity [24]. Topic 5 is related to integrin signaling pathways as well as several biosynthesis pathways. Topic 9 is involved with TGF-beta and AKT signaling transduction. Topic 10 is particularly interesting as it is associated with immune and inflammatory response genes and genesets. These include the top genes, *IL1RL1* and *IL5RA*, and the top genesets, *IL3* signaling pathway, *IL5* signaling pathway, and inflammatory response pathway. Indeed, previous studies have identified a relationship between AD treatment response, inflammation, and immune functioning [25, 26]. It is notable that several of the genes associated with differential methylation (*TRAPPC10*, *SLC1A5*, and *GPR19*) appear multiple times in the same topic, suggesting that top methylation sites are related. Additionally, *JUP* and *SLC2A5* were observed in both the top gene and top methylation sites of the same topic, which indicates a potential gene-methylation relationship within the topic.

### Association of top genes under AD-related topics with specific depressive symptoms

As the diagnosis of MDD requires the presence of any five diagnostic criteria out of a list of symptoms, provided that one of the criteria is presence of depressed mood or lack of interest, the symptoms used to diagnose depression may vary from individual to individual, we set out to determine if there were any molecular markers that associate with a given symptom. For each topic, we correlated the top 10 genes as described in the previous section with each individual MADRS item. The significant pairs after correction for multiple testing (BH-corrected FDR < 0.1) are listed in **S4 Table in** S2 File. Among the 10 MADRS responses, we found “reduced sleep”, “inner tension”, and “feeling” to be significantly associated with at least one gene. Three genes under topic 10 were significantly correlated with two MADRS responses at FDR < 0.05: *RAB44*—reduced sleep, *SEMA7A*—feeling, and *IL5RA*—feeling. At the more lenient threshold of FDR < 0.1, we found additional associations, including (in the format of topic-MADRS-gene(s)): topic 4—reduced sleep—*BIRC3*/*LIMS2*/*LDHB*/*ICOS*, topic 5—feeling—*GATA2*/*SLC45A3*, topic 5—reduced sleep—*GATA2*/*HDC*, topic 9—reduced sleep—*NR4A1*/*NEURL1*, topic 10—reduced sleep—*SMPD3*/*PTGDR2*/*PIK3R6*/*SEMA7A*/*IL1RL1*/*SLC7A8*/*CYP4F12*, and topic 10—feeling—*CYP4F12*/*PTGDR2*/*PI3KR6*/*SMPD3*/*RAB44*.

### Multi-omic quantitative trait loci analysis

To investigate the impact of genotype variation on AD response, we performed cis-eQTL and cis-mQTL analyses for the differential genes and methylation sites. We tested the top 10 genes under the AD-related topics and discovered several eQTL for genes *SLC45A3*, *ICAM4*, *PLXNB2*, and *PIK3R6*, which are among the top genes under topics 5, 9, 9, and 10, respectively (**S5 Table in** S2 File). Interestingly, for topic 9, we have identified multiple genes with cis-eQTLs in their regions, namely *ICAM4* and *PLXNB2*. Using the top methylation sites under each topic, we also identified a substantial number of mQTL (**S6 Table in** S2 File), implying epigenetic associations with AD-response.

The Manhattan plots in **S3 Fig in** S2 File show where genes and methylation sites are aligned by genomic location, identifying the in-cis relationships between the top features identified by the model. We found several top methylation sites that were proximal to one of the top genes with P-value < 0.05: Topic 4—*JUP*—cg11278727, topic 5—*SLC2A5*—cg04413644, topic 5—*SLC2A5*—cg25755851, topic 5—*MYB*—cg04531756, topic 10—*IL1RL1*—cg03938978, and topic 10—*CYP4F12*—cg24656834.

Finally, we performed regression analysis between the 18 top response SNPs, shown in Fig 2C (P-value < 10^−4^) and AD response-associated topics. The t-statistics of the regression analyses are shown as heatmaps in **S4 Fig in** S2 File. Not all SNPs are significantly correlated with the differential topics, and interestingly, each topic displayed a unique pattern of association with these SNPs. We identified 7 significant SNP-topic pairs, as shown in Table 1. Interestingly, several of these SNPs are located near genes which have previously been associated with MDD. Finally, in order to better characterize the effects of the minor allele, we grouped individuals who were heterozygous with those who were homozygous at the major allele and repeated this analysis. These results are shown in **S7 Table in** S2 File. The results in Table 1 and **S7 Table in** S2 File show different sets of SNPs. SNPs may affect a given phenotype in different ways. For example, a SNP may have different phenotypes with each of the three genotypes (homozygous major alleles, homozygous minor alleles, and heterozygous alleles). Alternatively, for some SNPs and their corresponding phenotype, homozygous major alleles and heterozygous alleles may show a similar phenotype. The minor allele frequency (MAF) model is aimed at capturing the dosage effect of an allele in the former case, while the presence / absence of major allele model is aimed at illustrating the latter scenario. We have identified different sets of SNPs from the two analyses, and it suggests that there are indeed different relationships between SNPs and the phenotypes. We focused our discussion on SNPs identified using the MAF model, but the alternative set of SNPs identified by presence / absence of the major allele could be interesting as well.

**Table 1:** Relationship between SNPs associated with response and response-associated topics. The associations between top SNPs and the response are indicated by the P-value (Res_pval). In cases where differential topics are associated with the SNPs (P-value < 0.1), P-value (Topic_pval) and correlation coefficient (Coef) are indicated. The chromosome (Chr) and genomic location (Pos) of each SNP are shown. The closest differentially expressed genes within 500kb are shown. Rows where SNPs are not associated with differential topics or have no proximal genes are denoted as NA.

SNP	Res_pval	Topic	Topic_pval	Coef	Chr	Pos	Gene
rs10917312	3.82E-05	4	0.0395	-0.1982	1	23152969	EPHB2
rs10917312	3.82E-05	9	0.0147	0.2338	1	23152969	EPHB2
rs2584937	3.52E-05	NA	NA	NA	2	24674385	SF3B6
rs11127134	9.11E-05	NA	NA	NA	2	28398324	NA
rs2738284	2.28E-05	NA	NA	NA	2	217311609	SMARCAL1
rs6783990	9.29E-05	4	0.0391	-0.1976	3	12797747	NA
rs17583756	6.74E-05	5	0.0548	0.1866	3	22004875	NA
rs17583756	6.74E-05	10	0.0476	-0.192	3	22004875	NA
rs7656458	8.49E-05	NA	NA	NA	4	125602470	NA
rs7772714	9.83E-06	4	0.0555	-0.1872	6	73334849	KCNQ5
rs2639	5.22E-05	9	0.042	-0.1952	7	6066461	USP42
rs4722385	8.20E-05	4	0.0139	-0.2362	7	24788209	CYCS
rs11062980	8.49E-05	10	0.0631	-0.1773	12	4150135	NA
exm977504	5.93E-05	9	0.0277	-0.2108	12	6562293	NCAPD2
rs12229174	7.25E-05	NA	NA	NA	12	64246666	NA
rs1874894	4.38E-06	NA	NA	NA	12	115886306	NA
rs4767329	2.67E-05	NA	NA	NA	12	115929439	NA
rs667973	4.16E-05	NA	NA	NA	13	101782678	NA
rs977045	2.85E-06	4	0.0961	-0.1611	16	76116063	ADAT1
rs977045	2.85E-06	9	0.0938	0.1622	16	76116063	ADAT1
rs6131220	6.25E-05	NA	NA	NA	20	11473176	NA

## Discussion

Currently, the molecular factors underlying response to AD treatment remain unknown impacting our capacity to better develop personalized treatment strategies for depressed patients, and develop new treatment avenues. While early studies investigated candidate genes or small panels of potential biomarkers, more recent studies have investigated genome-wide data, with the expectation that the combination of numerous biological measures may have a better capacity to predict or explain response. In the present study, we combined several genome-wide datasets in order to identify underlying biological characteristics related to AD response, as well as to identify biological pathways which may be relevant. Using the iGEM model, we identified four topics that were significantly associated with response. These topics comprised genes displaying differential expression between responders and non-responders, genes associated with DNA methylation differences, and genesets associated with these top genes. Interestingly, the topic that was most positively correlated with AD response was associated with immune and inflammatory functioning, both of which have been associated with MDD.

Several of the top genes identified using the model to examine gene expression and methylation data are related to depressive symptoms, and have previously been found to be differentially expressed in the context of depression or associated with AD response. In particular, we have identified IL-related genes interleukin 1 receptor like 1 (*IL1RL1*) and interleukin 5 receptor (*IL5*) subunit alpha (*IL5RA*) as well as the AKT/PIK3 pathway related gene phosphoinositide-3-kinase regulatory subunit 6 (*PIK3R6*). A previous study found that *IL5RA* and *PIK3R6* were upregulated in lithium-treated bipolar disorder patients [27]. *IL5* signaling affects the downstream AKT/PIK3 pathway, which suggests that AD response could be mediated in a top-down fashion from *IL5* to the AKT/PIK3 pathway and other downstream molecules [28]. *IL1RL1* mRNA was found to be differentially expressed in the spleen of mice with depression-like behaviour [29]. In addition, the expression of *IL1RL1* (also known as *ST2* and *IL33R*), has been shown to be negatively associated with depression severity in post-stroke depression, whereas the expression of its ligand, the cytokine *IL33*, was positively associated with severity [30–33]. In addition to the inflammatory regulators, another top gene in topic 10, sphingomyelin phosphodiesterase 3 (*SMPD3*), has also been associated with depression and AD treatment response. Acid sphingomyelinase (*ASM*) is a molecular target of AD drugs, and mice treated with the SSRI fluoxetine demonstrated inhibited *ASM* activity [34]. *ASM* could potentially mediate its effect through ceramide and sphingolipid-metabolizing enzymes including *SMPD3*, and *ASM* overexpression led to reduced *SMPD3* expression in mice, which was accompanied by an increase of depression-like behaviour [35, 36].

We included genotype information as a secondary set of analyses, and identified a number of SNPs which were related to response, including several which were also associated with the AD-related topics. Interestingly, several of the genes proximal to these SNPs have previously been associated with depression, including nuclear receptor coactivator 1 (*NCOA1*), which is 40398 bp downstream of rs2584937 [37]. *NCOA1* is involved in glucocorticoid receptor signaling, and impairments of glucocorticoid receptor signaling have previously been associated with MDD [38]. Moreover, the increase of *NCOA1* during pregnancy was diminished in depressed patients, which also suggests an association between depression and *NCOA1* [39]. Also of note is the relationship between response and exm977504 (rs2041385), which is found within TAP Binding Protein Like (*TAPBPL*), an MHC-related gene which has previously been shown to be influenced by glucocorticoid signaling [40]. This SNP is an eQTL for *TAPBPL* expression in various tissues including blood, brain, LCL, and monocytes [37, 41–44]. Interestingly, additional eQTLs were found for TAPBPL in an MDD population, however this association was only significant in females [40]. We also identified SNPs which were near genes which were differentially expressed in relation to AD response, and which have previously been associated with MDD. rs10917312 is near a member of receptor tyrosine kinase transmembrane glycoproteins, EPH Receptor B2 (*EPHB2*). KO mice with *EPHB2* deficiency have display depressive and stressed behaviours [45, 46]. Additionally, the deficiency in human APP/PS1 transgenic mice can be alleviated with overexpression of *EPHB2* with improved impaired memory, depression, and anxiety-like behaviours [47]. Another gene to be noted is Potassium Voltage-Gated Channel Subfamily Q Member 5 (*KCNQ5*), which is located near rs7772714. *KCNQ5* was downregulated along with several other genes in the cholinergic synapse pathway in individuals with MDD [48]. Furthermore, the expression of *KCNQ5* was upregulated in mice treated with amitriptyline, which indicates a potential role of signaling transduction in the AD response [49]. Although we did not identify in-cis associations between the top response SNPs and the top features in differential topics, the topic-SNP-gene relationships identified from our analyses complement our findings with iGEM, and shed light on the complex relation between various genes associated with MDD pathogenesis and AD response.

The proposed iGEM model has several advantages. As previously mentioned, AD response in MDD is a highly complex biological process, that likely involves, and is influenced by, numerous genetic and epigenetic processes. One of the advantages of the model is that it promotes the finding of multiple features with similar functions by integrating geneset information. In addition to gene expression and DNA methylation, the model is flexible and may accept different types of biological readings. A potential usage may include metabolite readings. If there are known associations between metabolites and a particular disease, these can be included in a similar fashion as the geneset information in the current model. There are several limitations of the model that must be noted. Because our sample size is small, we opted to choose genes or methylation sites based on the marginal p-values without multiple testing correction. If we correct the gene expression and methylation for multiple testing, no features pass an FDR threshold of 0.01. Analyses with SNPs were also limited by the small sample size, therefore multiple testing corrections were not performed globally. Indeed, this was the main motivation for our study because we represent the high-dimensional features using the low-dimensional iGEM-derived topics to improve the statistical power. Also, we used the same dataset twice–once for feature selection and once for iGEM model training. This may introduce ascertainment biases into the analysis. Ideally, for a much larger dataset, we could have split the data into two sets, one for choosing differential genes and CpG sites and one for training our iGEM using those pre-selected features. Given the small sample size, we opted to use the same dataset to do the pre-screening and model training. We were aware of the limitation, especially when considering the performance of patient clustering based on patient factor score with the same dataset. Nonetheless, we found that the power we lack in sample numbers was compensated by the integration of multiple modalities, which we were able to leverage through our model. The fact that certain groups of genes and CpG sites gained higher score in a response related topic suggests their potential roles in AD response. Currently, the model only supports two types of data input, but has the potential to extend to three or more types if needed. Another limitation of the model is that, as an NMF-based model, it takes only non-negative inputs. Therefore, it may be difficult to apply the model to certain data types that contain negative values, which requires proper normalization before applying the model.

In summary, we found biologically meaningful features related to MDD and AD response, which may ultimately lead to not only the ability to predict AD response, but also the development of new treatments for MDD. With the auxiliary geneset information, the model promotes findings of biological relevance and intuitive interpretation of latent topic based on the associated genesets. For example, the immune and inflammatory processes highlighted by topic 10, as well as the association of several response-associated SNPs with genes and pathways which have previously been associated with MDD.

Overall, the model identified several biological differences between individuals who responded to AD treatment and those who did not, and emphasizes the involvement of immune system in AD treatment response. Future studies are needed to better characterize the role of these molecular mechanisms in AD response, as well as to determine how well these findings extend to other cohorts.

## Materials and methods

### Data

#### Patient cohort

Participants were recruited from an outpatient clinic at the Douglas Mental Health University Institute in Montréal, Canada, between February 2012 and October 2016. All individuals were diagnosed with major depressive disorder, and had an active episode when recruited. Exclusion criteria included comorbidity with other major psychiatric disorders, bipolar disorder, alcohol or substance abuse over the last 6 months, and a concurrent physical illness. All subjects were free of psychotropic medication at baseline (T0). Patients were treated for 8 weeks (T8) with either i) Desvenlafaxine, a serotonin–norepinephrine reuptake inhibitor, started at 50 mg die and increased to 100 mg if needed; or ii) Escitalopram, started at 10mg die and increased to 20mg if needed. All subjects were assessed at baseline and after 8 weeks (T8) using the Montgomery–Åsberg Depression Rating Scale (MADRS), and were classified as responders (RES) (N = 83) or non-responders (NRES) (N = 79) based on a ≥ 50% reduction in scores from baseline [50]. This study was approved by our local institutional review board, and all participants provided written informed consent. Only patients with data available for gene expression, methylation, genotype, and MADRS response at both week 0 and week 8 were included, giving a final subject count of 61 RES and 50 NRES (**S1 Table in** S2 File).

#### Blood samples

Peripheral blood samples were collected at baseline, and tubes were frozen using a sequential freezing process. DNA was extracted from PAXgene DNA tubes (PreAnalytix) using a modified version of the Qiagen FlexiGene DNA kit, and was stored at -20°C. Whole blood for RNA was collected in EDTA tubes and filtered using LeukoLOCK filters (Life Technologies). Total RNA was extracted using a modified version of the LeukoLOCK Total RNA Isolation System protocol, and included DNase treatment to remove genomic DNA. RNA quality was assessed using the Agilent 2200 Tapestation, and only samples with RNA Integrity Number (RIN) ≥ 6.0 were used.

#### Gene expression

RNA sequencing was performed using RNA extracted at baseline for 150 samples (79 responders, 71 non-responders). All libraries were prepared using the Illumina TruSeq mRNA stranded protocol following the manufacturer’s instructions. Samples were sequenced at the McGill University and Genome Quebec Innovation Centre (Montreal, Canada) using the Illumina HiSeq4000 with 75nt paired-end reads. FASTXToolkit and Trimmomatic were used for quality and adapter trimming, respectively [51, 52]. Tophat2, using bowtie2, was used to align the cleaned reads to the reference genome (GRCh38) [53, 54]. Reads that lost their mates through the cleaning process were aligned independently from the reads that still had pairs. Quantification on each gene’s expression was estimated using HTSeq-count and a reference transcript annotation from ENSEMBL [55]. Counts for the paired and orphaned reads for each sample were added to each other. Normalization was conducted on the resulting gene matrix using DESeq2 [56]. Normalized counts were further log 2 transformed and corrected for age, sex, and RIN using the removeBatchEffect function of limma [57]. Genes with counts lower than 30 were filtered. A total of 58126 genes passed the filter and were then applied to the subsequent differential analysis.

#### Methylation

DNA extracted at baseline for 147 samples (71 responders, 76 non-responders) was bisulfite treated using the EZ DNA Methylation-Gold Kit (Zymo Research), and hybridized to the Infinium MethylationEPIC Beadchip (Illumina). The Infinium MethylationEPIC Beadchip was used to assess genome-wide DNA methylation (Illumina, US). After accounting for attrition rates, and DNA sample quality control, pre-processing and analysis of raw microarray data for the remaining samples was conducted within R (version 3.4) predominantly using the Chip Analysis Methylation Pipeline (ChAMP) Bioconductor package, which utilizes many elements of minfi [58, 59]. Sample methylation signal QC was assessed by plotting log median methylated and unmethylated signals. Samples were removed if they failed to cluster with others or if they exhibited lower median intensities in either signal channel. Probes with low signal detection relative to control probes, probes with < 3 beads in > 5% of samples, cross reactive probes, non-CpG probes, sex chromosome probes, and probes that hybridize to single nucleotide polymorphism sites (SNPs) were removed. Beta values were calculated as the ratio of methylated signal to the sum of unmethylated and methylated signals at each CpG site, and subsequently normalized. Log2 transformed beta values were used for the remainder of pre-processing steps as recommended by Du et al., but reported as beta values [60]. Next, the singular value decomposition (SVD) method was called by *champ*.*SVD* in order to assess the amount and significance of technical batch components, along with any potential confounding variables, in our dataset. Using the *champ*.*runCombat* function, Combat algorithms were applied in order to correct for our two initial submission batches, along with slide and array as technical batch components detected by SVD. Combat relies on parametric empirical Bayes frameworks when adjusting data for batch effects in a manner suitable for larger sample sizes [61]. Age and sex were corrected for as covariates after being identified as confounding biological components through SVD. The CpG site annotations are based on the chip manifest (the manifest uses information from the UCSC) [53]. CpG sites are annotated to a gene if they are in the body or less than 1500bp upstream of the transcription start site (TSS). Detected and known batch effects were corrected for prior to differential methylation analysis. We used DNA methylation beta values from 673039 methylation sites for the differential analysis.

#### Genotype

Genotyping was performed for 157 subjects (76 responders, 81 non-responders) using the Infinium PsychArray Beadchip (Illumina). The genotype information from a total of 598131 SNPs were then filtered through PLINK [62]. The SNP variations were encoded as 0, 1, and 2.

#### Depression scores

We used both total MADRS scores, as well as scores from individual items. Scores for each item range between 0 to 6, with higher scores indicating more severe symptoms. For use in the model, we defined the total score as the difference between total scores at week 0 and week 8, divided by of the total score at week 0. The MADRS questionnaire includes 10 items covering sadness (reported sadness and apparent sadness), negative thoughts (pessimistic and suicidal), detachment (concentration, lassitude, feeling), and neurovegetative symptoms (inner tension, reduced sleep, and reduced appetite) [63]. Sadness includes reported sadness and apparent sadness. Negative thoughts include pessimistic thoughts and suicidal thoughts. Inner tension, reduced sleep, and reduced appetite belong to the neurovegetative symptoms. Detachment covers concentration difficulties, lassitude, and inability to feel.

#### Genesets

An integrated human geneset dataset with a total of 4337 Genesets was adopted from the Bader lab (https://baderlab.org/GeneSets). Similar genesets were merged together recursively to create a merged geneset if the smaller / larger geneset contains at least 50% / 80% overlapped genes. A total of 1087 genesets were applied to the model after the merging. The geneset were then normalized over genes to create the geneset-gene matrix ρ.

### Single-omic differential analyses

The gene expression level and methylation beta value readings were corrected for age and sex and normalized by mean. To balance the difference in feature number between different omics, for each omic, the inputs were normalized by multiplying a ratio of the sum of feature number of other omics over the sum of feature number of all omics. In a 2-omic setting, say if 2 omics X1 and X2 have a number of n1 and n2 features, the model input is multiplied by a factor of n2/(n1+n2) and n1/(n1+n2) respectively.

#### Expression analysis

Differential genes included in the Bader geneset were selected by DESeq2 with P < 0.05 [56]. A total of 1572 differential genes were selected.

#### Methylation analysis

Differential methylation sites proximal to at least one of the selected genes (within 500KBP) were selected with average beta value difference > 0.025 between RES and NRES. A total of 13900 differential methylation sites were selected.

#### Gene-methylation site interaction

We selected genes which were correlated with proximal methylation sites within 500KBP by Pearson correlation. For each gene, the top 10 pairs with P < 0.05 were considered as valid interaction pairs and were used to construct the interaction adjacency matrix B described in the model derivation section between gene expression and methylation.

#### Genotype analysis

Differential SNPs were identified through PLINK. A filter of Hardy-Weinberg equilibrium tests at a threshold of 0.001, missing call rate threshold at 0.01 for each SNP, and MAF threshold at 0.05 were applied, resulting in 263740 SNPs in our analysis. The SNPs were correlated with AD response and corrected by age and sex by ordinary least squares regression analysis. PCA was performed with 10 PCs. The top two PCs explain small proportions of variance, namely 3.3% and 2.1%, respectively. Given the low variance ratio, we did not correct for PCs for the following analyses.

### Multi-omic analyses

#### Geneset-aware NMF

To integrate the multiomic data using prior gene set information, we developed a non-negative matrix factorization (NMF) approach called geneset-aware integrative Geneset-Embedded NMF (iGEM). iGEM uses gene set information as the predefined basis matrices to help identify enriched gene sets from the gene expression and DNA methylation data. We applied iGEM to the differential genes and methylation sites selected from the single-omic differential analyse. We applied the model to derive 10 topics and the corresponding factor scores for the downstream analyses. The model design is described in the S2 File.

#### Correlation analysis

Spearman correlation was performed between topic factor score W and patient condition y. Pearson correlation was performed between topic factor score W and MADRS scores. For each topic, the gene expression level and the methylation beta value from the top 10 features were correlated with MADRS score by Pearson correlation. The correlations were corrected by the Benjamini-Hochberg (BH) procedure with FDR < 0.05.

#### QTL analysis

QTL analyses were performed for the genes and methylation sites based on cis-SNPs within 500kb. In addition to gene expression and DNA methylation, QTL analysis was performed for the 10 MADRS responses and 10 topics. The QTL analyses were corrected by the BH procedure with FDR < 0.1 over in-cis SNPs.

#### Top response SNP analysis

To investigate the role of genotype variation in MDD and AD response, we have selected SNPs that were highly correlated to response with P-value < 10^−4^. Similar to the analysis previously described in the genotype analysis section, we performed regression analysis between genotype variation and patient factor score. Two sets of analyses were performed for genotype coded as either the number of minor alleles (0, 1, or 2), or separating patients by presence or absence of the major allele. The former was performed to investigate the potential for additive effects of a variant, while the latter investigates the potential dominant effect of an allele.

## Supporting information

S1 File (ZIP)

S2 File (DOCX)
