# Supplementary material for: Multi-omic modeling of antidepressant response implicates dynamic immune and inflammatory changes in individuals who respond to treatment
Source: PLoS One. 2023 May 15;18(5):e0285123. doi: 10.1371/journal.pone.0285123 (PMC10184917; doi:10.1371/journal.pone.0285123)
Supplement: S2 File — (DOCX) [file pone.0285123.s002.docx]

## Integrative Geneset-Embedded non-negative Matrix factorization (iGEM)

### Model Loss

With the squared error loss function L, a number of k multi-omic datasets can be denoted as X (patient by feature) and reconstructed as matrices patient factor score W (patient by topic) and feature factor score H (topic by feature), given n features, m topics, and p patients. e is a vector of ones / a matrix with ones on the diagonal and zeros elsewhere. The sparsity constraint is performed as the square of L1-norm on the column vector h_j_, which is the j-th column of H. Here, we denote the squared error loss and the sparsity constraint as L_a_.

$$L_{a} = \sum_{k} \left\| X_{k}-{WH}_{k} \right\|_{F}^{2}+\gamma_{k}\sum_{n} \left\| h_{kn} \right\|_{1}^{2}$$

$$= \sum_{k} \left\| X_{k}-{WH}_{k} \right\|_{F}^{2}+ \gamma_{k}\sum_{n} \left( \sum_{m} H_{k}\left( m,n \right) \right)^{2}$$

$$= \sum_{k} \left\| \left( \begin{matrix} W \\ \sqrt{\gamma_{k}}e_{1xm} \end{matrix} \right)H_{k}\left( \begin{matrix} X_{k} \\ 0_{1xn_{k}} \end{matrix} \right) \right\|_{F}^{2}$$

$$= \sum_{k} Tr(X_{k}X_{k}^{T}) - 2Tr(X_{k}H_{k}^{T}W^{T}) + Tr(WH_{k}H_{k}^{T}W^{T}) + Tr(\gamma_{k}e_{1xm}H_{k}H_{k}^{T}e_{mx1})$$

$$\frac{\partial L_{a}}{\partial H_{k}} = -2W^{T}X_{k} + 2W^{T}WH_{k} + 2\gamma_{k}e_{mxm}H_{k}$$

In addition to the sparsity constraint, a rewarding term can be added to strengthen the entries involving the interaction within each dataset or between different datasets. The rewarding terms, with the strength determined by the coefficients λ_k_ and λ_kj_, are added as a trace between the feature factor scores H and the adjacency matrix. The adjacency matrices A and B indicate the interactions within H_k_ and between H_k_ and another dataset H_j_ respectively. For the patient factor score W, a penalty term ${Ω\left\| W \right\|}_{F}^{2}$ is added to limit its increase. We combine these constraints as L_b_.

$$L_{b} = -\sum_{k} \lambda_{k}Tr(H_{k}AH_{k}^{T}) - \sum_{j}^{j\neq k} \lambda_{kj}Tr(H_{k}BH_{j}^{T}) + {Ω\left\| W \right\|}_{F}^{2}$$

$$\frac{\partial L_{b}}{\partial H_{k}} = -2\lambda_{k}H_{k}A - \sum_{j}^{j\neq k} \lambda_{kj}H_{j}B^{T}$$

For a dataset such as gene expression with additional information of gene composition in genesets, it is possible to derive gene and geneset information with shared topic. The matrix H (topic by gene) can be reconstructed by matrices α (topic by geneset) and ρ (geneset by gene), with a number of l genesets. The squared error loss function L_c_ with sparsity constraint on α can be written in a similar fashion as L_a_.

$$L_{c} = \sum_{k} \left\| H_{k}-{\alpha_{k}\rho}_{k} \right\|_{F}^{2}+\gamma_{k}\sum_{l} \left\| \alpha_{kl} \right\|_{1}^{2}$$

$$= \sum_{k} Tr(H_{k}H_{k}^{T}) - 2Tr(H_{k}\rho_{k}^{T}\alpha_{k}^{T}) + Tr({\alpha_{k}\rho}_{k}\rho_{k}^{T}\alpha_{k}^{T}) + Tr(\gamma_{k}e_{1xm}\alpha_{k}\alpha_{k}^{T}e_{mx1})$$

The loss function of the model L is composed of L_a_, L_b_, and L_c_:

$$L = L_{a} + L_{b} + L_{c}$$

$$= \sum_{k} Tr(X_{k}X_{k}^{T}) - 2Tr(X_{k}H_{k}^{T}W^{T}) + Tr(WH_{k}H_{k}^{T}W^{T}) + Tr(\gamma_{k}e_{1xm}H_{k}H_{k}^{T}e_{mx1}- \sum_{k} \lambda_{k}Tr(H_{k}AH_{k}^{T}) - \sum_{j}^{j\neq k} \lambda_{kj}Tr(H_{k}BH_{j}^{T}) + {Ω\left\| W \right\|}_{F}^{2} + \sum_{k} Tr(H_{k}H_{k}^{T}) - 2Tr(H_{k}\rho_{k}^{T}\alpha_{k}^{T}) + Tr({\alpha_{k}\rho}_{k}\rho_{k}^{T}\alpha_{k}^{T}) + Tr(\gamma_{k}e_{1xm}\alpha_{k}\alpha_{k}^{T}e_{mx1})$$

### Multiplicative Update

For the patient factor score W, the partial derivative of L with respective to W can be written as

$\frac{\partial L}{\partial W} = \sum_{k} X_{k}H_{k}^{T} + WH_{k}H_{k}^{T}+ ΩW$. Note that a common numerical constant of 2 are removed for brevity, and the same will be applied to the rest of the partial derivative steps. The multiplicative update of W can be performed with a learning rate $\eta_{k} = \frac{W}{WH_{k}H_{k}^{T}+ ΩW}$.

$$W_{pm} ⃪ \sum_{k} W_{pm}-\eta_{kpm}\left( -X_{k}H_{k}^{T}+WH_{k}H_{k}^{T}+ΩW \right)$$

$$= W_{pm}\sum_{k} \left( \frac{X_{k}H_{k}^{T}-WH_{k}H_{k}^{T} - ΩW+WH_{k}H_{k}^{T}+ΩW}{WH_{k}H_{k}^{T}+ ΩW} \right)_{pm}$$

$$= W_{pm}\sum_{k} \left( \frac{X_{k}H_{k}^{T}}{WH_{k}H_{k}^{T}+ ΩW} \right)_{pm}$$

For a geneset factor score α_k_, the partial derivative of L with respective to α_k_ can be written as

$\frac{\partial L}{\partial\alpha_{k}} = -H_{k}\rho_{k}^{T} + {\alpha_{k}\rho}_{k}\rho_{k}^{T} + \gamma_{k}e_{mxm}\alpha_{k}$. The multiplicative update of α_k_ can be performed with a learning rate $\eta_{k} = \frac{\alpha_{k}}{{\alpha_{k}\rho}_{k}\rho_{k}^{T} + \gamma_{k}e_{mxm}\alpha_{k}}$.

$$\alpha_{kml} ⃪ \alpha_{kml}-\eta_{kml}\left( -H_{k}\rho_{k}^{T} + {\alpha_{k}\rho}_{k}\rho_{k}^{T} + \gamma_{k}e_{mxm}\alpha_{k} \right)_{ml}$$

$$= \alpha_{kml}{(\frac{H_{k}\rho_{k}^{T}}{{\alpha_{k}\rho}_{k}\rho_{k}^{T} + \gamma_{k}e_{mxm}\alpha_{k}})}_{ml}$$

For a feature factor score H_k_, the partial derivative of L with respective to H_k_ can be written as

$\frac{\partial L}{\partial H_{k}} = -{W^{T}X}_{k} + W^{T}WH_{k} + \gamma_{k}e_{mxm}H_{k} - \lambda_{k}H_{k}A - \sum_{j}^{j\neq k} 0.5\lambda_{kj}H_{j}B^{T} + H_{k} - {\alpha_{k}\rho}_{k}$. The multiplicative update of H_k_ can be performed with a learning rate $\eta_{k} = \frac{H_{k}}{W^{T}WH_{k} + \gamma_{k}e_{mxm}H_{k} + H_{k}}$.

$$H_{kmn} ⃪ H_{kmn}-\eta_{kmn}\left( -{W^{T}X}_{k} + W^{T}WH_{k} + \gamma_{k}e_{mxm}H_{k} - \lambda_{k}H_{k}A - \sum_{j}^{j\neq k} 0.5\lambda_{kj}H_{j}B^{T} + H_{k} - {\alpha_{k}\rho}_{k} \right)_{ml}$$

$$= H_{kmn}{(\frac{{W^{T}X}_{k} + \lambda_{k}H_{k}A + \sum_{j}^{j\neq k} 0.5\lambda_{kj}H_{j}B^{T} + {\alpha_{k}\rho}_{k}}{W^{T}WH_{k} + \gamma_{k}e_{mxm}H_{k} + H_{k}})}_{mn}$$

### Model update steps

1. Initialize W, H, and α.

2. Fix H and update W with ${W_{pm} ⃪ W}_{pm}\sum_{k} \left( \frac{X_{k}H_{k}^{T}}{WH_{k}H_{k}^{T}+ ΩW} \right)_{pm}$

(Solve ${min}_{W\geq0}\sum_{k} \left\| X_{k}-{WH}_{k} \right\|_{F}^{2} +{Ω\left\| W \right\|}_{F}^{2}$)

3. Fix W, α and update H with $H_{kmn} ⃪ H_{kmn}{(\frac{{W^{T}X}_{k} + \lambda_{k}H_{k}A + \sum_{j}^{j\neq k} 0.5\lambda_{kj}H_{j}B^{T} + {\alpha_{k}\rho}_{k}}{W^{T}WH_{k} + \gamma_{k}e_{mxm}H_{k} + H_{k}})}_{mn}$

(Solve ${min}_{H\geq0}\sum_{k} \left\| X_{k}-{WH}_{k} \right\|_{F}^{2} +\gamma_{k}\sum_{n} \left\| h_{kn} \right\|_{1}^{2} - \lambda_{k}Tr(H_{k}AH_{k}^{T}) - \sum_{j}^{j\neq k} \lambda_{kj}Tr(H_{k}BH_{j}^{T}) + \sum_{k} \left\| H_{k}-{\alpha_{k}\rho}_{k} \right\|_{F}^{2}$)

4. Fix H and update α with $\alpha_{kml} ⃪ \alpha_{kml}{(\frac{H_{k}\rho_{k}^{T}}{{\alpha_{k}\rho}_{k}\rho_{k}^{T} + \gamma_{k}e_{mxm}\alpha_{k}})}_{ml}$

(Solve ${min}_{\alpha\geq0}\sum_{k} \left\| H_{k}-{\alpha_{k}\rho}_{k} \right\|_{F}^{2} +\gamma_{k}\sum_{l} \left\| \alpha_{kl} \right\|_{1}^{2}$)

5. Repeat 2-4 until convergence or reaching maximum number of iterations.

### Model settings

There are several hyperparameters that affect the model performance. The reward term between omics λ_kj_ is set at 0.01. The penalty term Ω for patient factor score W is set at 2E^-6^. The sparsity constraint γ for gene, geneset, and methylation are set at 0.1, 0.05, and 0.01 respectively.

Supp. Table 1 Demographic characteristics of cohort. Individuals with major depressive disorder were classified as treatment non-responders (NRES) or responders (RES) based on a ≤ 50% reduction in total Montgomery-Åsberg Depression Rating Scale (MADRS) score between baseline (T0) and after 8 weeks of treatment (T8) with either escitalopram or desvenlafaxine. Values are reported as average ± standard error.

|  | **NRES** | **RES** |
| --- | --- | --- |
| N | 50 | 61 |
| Age | 41.0 ± 1.8 | 38.6 ± 1.6 |
| Sex (% female) | 60.0% | 65.6% |
| Medication (% escitalopram) | 56.0% | 44.3% |
| MADRS (T0) | 34.2 ± 0.8 | 31.9 ± 0.8 |
| MADRS (T8) | 26.5 ± 1.1 | 8.9 ± 0.7 |

Supp. Table 2. Spearman correlation between the patient factor score and the patient condition. Each row represents the Spearman correlation coefficient (Coef.), P-value, and corrected P-value after multiple testing (BH) of a topic.

| Topic | Coef. | P-value | BH |
| --- | --- | --- | --- |
| 4 | 0.3165 | 0.0007 | 0.0071 |
| 10 | -0.2729 | 0.0038 | 0.0188 |
| 9 | -0.2577 | 0.0063 | 0.0211 |
| 5 | -0.2283 | 0.016 | 0.0399 |
| 6 | -0.1916 | 0.044 | 0.088 |
| 2 | -0.1503 | 0.1153 | 0.1922 |
| 3 | -0.1368 | 0.1524 | 0.2177 |
| 1 | 0.1051 | 0.2722 | 0.3403 |
| 8 | 0.022 | 0.8184 | 0.9093 |
| 7 | 0.0085 | 0.9296 | 0.9296 |

­

Supp. Table 3. Pearson correlation between the patient factor score and the MADRS response. Each row represents the Pearson correlation coefficient (Coef.), P-value, and corrected P-value after multiple testing between a MADRS response and a topic.

| MADRS | Coef. | P-value | | BH | Topic |
| --- | --- | --- | --- | --- | --- |
| feeling | 0.3571 | 0.0001 | 0.0012 | | 10 |
| reduced_sleep | 0.3335 | 0.0003 | 0.0017 | | 10 |
| reduced_sleep | -0.2531 | 0.0074 | 0.0736 | | 4 |
| inner_tension | 0.2469 | 0.009 | 0.0899 | | 9 |
| lassitude | 0.2242 | 0.018 | 0.1799 | | 6 |
| reported_sadness | 0.2195 | 0.0206 | 0.1091 | | 5 |
| apparent_sadness | 0.2175 | 0.0218 | 0.1091 | | 5 |
| apparent_sadness | -0.202 | 0.0335 | 0.1001 | | 4 |
| feeling | -0.2007 | 0.0347 | 0.1001 | | 4 |
| reported_sadness | -0.1952 | 0.0401 | 0.1001 | | 4 |

Supp. Table 4. Pearson Correlation between the gene factor score and the MADRS item response. Each row represents the Pearson correlation coefficient (Coef.), P-value, and corrected P-value after multiple testing between a top gene from the corresponding topic and MADRS response.

| Gene | MADRS item | Coef. | P-value | BH | Topic |
| --- | --- | --- | --- | --- | --- |
| DYNLT3 | reduced_sleep | -0.2658 | 0.0048 | 0.0585 | 1 |
| LMBRD1 | inner_tension | -0.3502 | 0.0002 | 0.059 | 1 |
| HAT1 | reduced_sleep | -0.2298 | 0.0152 | 0.0892 | 1 |
| SCOC | reduced_sleep | -0.2208 | 0.0199 | 0.0953 | 1 |
| ATG4C | reduced_sleep | -0.2173 | 0.0219 | 0.0972 | 1 |
| HAT1 | inner_tension | -0.2612 | 0.0056 | 0.0985 | 1 |
| MYOF | reduced_sleep | 0.2213 | 0.0196 | 0.0948 | 3 |
| BIRC3 | reduced_sleep | -0.2909 | 0.002 | 0.0503 | 4 |
| LIMS2 | reduced_sleep | -0.243 | 0.0102 | 0.0784 | 4 |
| LDHB | reduced_sleep | -0.2378 | 0.012 | 0.0818 | 4 |
| ICOS | reduced_sleep | -0.2224 | 0.019 | 0.0943 | 4 |
| SLC45A3 | feeling | 0.3005 | 0.0014 | 0.0789 | 5 |
| GATA2 | reduced_sleep | 0.2394 | 0.0114 | 0.0809 | 5 |
| HDC | reduced_sleep | 0.2318 | 0.0144 | 0.0864 | 5 |
| GATA2 | feeling | 0.2872 | 0.0022 | 0.092 | 5 |
| HERC1 | reduced_sleep | 0.2523 | 0.0076 | 0.0698 | 6 |
| DYNC1H1 | reduced_sleep | 0.2179 | 0.0216 | 0.0972 | 6 |
| BIRC6 | reduced_sleep | 0.2186 | 0.0212 | 0.0972 | 6 |
| TNK1 | reduced_sleep | -0.2393 | 0.0114 | 0.081 | 7 |
| ENO2 | reduced_sleep | -0.2279 | 0.0161 | 0.0907 | 7 |
| CRISPLD2 | feeling | 0.3072 | 0.001 | 0.0786 | 8 |
| UBE2B | reduced_sleep | -0.2164 | 0.0225 | 0.0983 | 8 |
| NR4A1 | reduced_sleep | 0.2213 | 0.0196 | 0.0948 | 9 |
| NEURL1 | reduced_sleep | 0.2179 | 0.0216 | 0.0972 | 9 |
| RAB44 | reduced_sleep | 0.3484 | 0.0002 | 0.0377 | 10 |
| SEMA7A | feeling | 0.3478 | 0.0002 | 0.0413 | 10 |
| IL5RA | reduced_sleep | 0.3026 | 0.0012 | 0.0442 | 10 |
| SMPD3 | reduced_sleep | 0.2845 | 0.0025 | 0.0519 | 10 |
| PTGDR2 | reduced_sleep | 0.2733 | 0.0037 | 0.0577 | 10 |
| PIK3R6 | reduced_sleep | 0.2669 | 0.0046 | 0.0583 | 10 |
| CYP4F12 | feeling | 0.3276 | 0.0004 | 0.0641 | 10 |
| SEMA7A | reduced_sleep | 0.2546 | 0.007 | 0.0689 | 10 |
| PTGDR2 | feeling | 0.3028 | 0.0012 | 0.0789 | 10 |
| IL1RL1 | reduced_sleep | 0.2387 | 0.0116 | 0.0813 | 10 |
| PIK3R6 | feeling | 0.2914 | 0.0019 | 0.0891 | 10 |
| SLC7A8 | reduced_sleep | 0.23 | 0.0152 | 0.0892 | 10 |
| SMPD3 | feeling | 0.2902 | 0.002 | 0.09 | 10 |
| RAB44 | feeling | 0.284 | 0.0025 | 0.092 | 10 |
| CYP4F12 | reduced_sleep | 0.2177 | 0.0217 | 0.0972 | 10 |

Supp. Table 5. eQTL Result of top 10 genes in the differential topics. The columns include the topic, the chromosome (Chr), the gene name and its genomic location (Gene, Gene_Start, Gene_End), the SNP name and its genomic location (SNP, SNP_Loc), QTL correlation statistics including P-Value, correlation coefficient, and BH-corrected FDR (P-value, Coef, BH) were included.

| Topic | Chr | Gene | Gene_Start | Gene_End | SNP | SNP_Loc | P-value | Coef | BH |
| --- | --- | --- | --- | --- | --- | --- | --- | --- | --- |
| 5 | 1 | SLC45A3 | 205626979 | 205649637 | rs913722 | 205318983 | <0.0001 | -0.3115 | 0.0042 |
| 9 | 19 | ICAM4 | 10397631 | 10399198 | rs875569 | 10668953 | 0.0026 | 0.2048 | 0.0928 |
| 9 | 19 | ICAM4 | 10397631 | 10399198 | exm1426306 | 10670992 | 0.0021 | 0.2115 | 0.0928 |
| 9 | 19 | ICAM4 | 10397631 | 10399198 | rs12984043 | 10672493 | 0.0003 | 0.2609 | 0.0331 |
| 9 | 22 | PLXNB2 | 50713408 | 50746075 | exm2235942 | 50713409 | 0.0011 | -0.2123 | 0.0866 |
| 9 | 22 | PLXNB2 | 50713408 | 50746075 | exm1621884 | 50716167 | 0.0002 | -0.2403 | 0.0283 |
| 10 | 17 | PIK3R6 | 8706040 | 8770994 | rs6503145 | 8613462 | 0.0019 | 0.2148 | 0.0848 |
| 10 | 17 | PIK3R6 | 8706040 | 8770994 | rs2101938 | 8620629 | 0.0015 | -0.2386 | 0.0848 |
| 10 | 17 | PIK3R6 | 8706040 | 8770994 | rs8071193 | 8655415 | 0.0015 | 0.2209 | 0.0848 |

Supp. Table 6. mQTL Result of top 10 methylation sites in the differential topics. The columns include the topic, the chromosome (Chr), the methylation site name and its genomic location (Met, Met_Start), the SNP name and its genomic location (SNP, SNP_Loc), QTL correlation statistics including P-Value, correlation coefficient, and BH-corrected FDR (P-value, Coef, BH) were included. The closest proximal gene to the methylation site and SNP were also included (Met_Gene and SNP_Gene). Rows where SNPs are not proximal to genes are denoted as NA.

| Topic | Chr | Met | Met_Start | SNP | SNP_Loc | Met_Gene | SNP_Gene | P-value | Coef | BH |
| --- | --- | --- | --- | --- | --- | --- | --- | --- | --- | --- |
| 4 | 3 | cg05107650 | 141441554 | rs1026716 | 140983891 | GRK7 | NA | 0.0051 | 0.0521 | 0.0875 |
| 4 | 3 | cg05107650 | 141441554 | psy_rs12491228 | 141031422 | GRK7 | GRK7 | 0.0069 | 0.0593 | 0.0989 |
| 4 | 3 | cg05107650 | 141441554 | rs9825379 | 141137035 | GRK7 | GRK7 | 0.0004 | 0.1089 | 0.0189 |
| 4 | 3 | cg05107650 | 141441554 | exm-rs10513137 | 141143430 | GRK7 | GRK7 | 0.0023 | 0.0880 | 0.0659 |
| 4 | 3 | cg05107650 | 141441554 | rs6762826 | 141145315 | GRK7 | GRK7 | 0.0045 | 0.0783 | 0.0875 |
| 4 | 3 | cg05107650 | 141441554 | rs295322 | 141326602 | GRK7 | GRK7 | 0.0001 | 0.1144 | 0.0046 |
| 4 | 16 | cg09821790 | 68283575 | psy_rs77517043 | 68301410 | NFATC3 | NFATC3 | 0.0069 | 0.0319 | 0.0500 |
| 4 | 16 | cg09821790 | 68283575 | rs9935025 | 68307447 | NFATC3 | NFATC3 | 0.0038 | -0.0201 | 0.0363 |
| 4 | 16 | cg09821790 | 68283575 | psy_rs55757091 | 68323654 | NFATC3 | NFATC3 | 0.0020 | 0.0297 | 0.0310 |
| 4 | 16 | cg09821790 | 68283575 | exm1251596 | 68344696 | NFATC3 | SMPD3 | 0.0028 | 0.0201 | 0.0329 |
| 4 | 16 | cg09821790 | 68283575 | rs13336173 | 68345691 | NFATC3 | SMPD3 | 0.0006 | 0.0233 | 0.0229 |
| 4 | 16 | cg09821790 | 68283575 | rs7189887 | 68353915 | NFATC3 | SMPD3 | 0.0066 | 0.0288 | 0.0500 |
| 4 | 16 | cg09821790 | 68283575 | rs1868158 | 68398924 | NFATC3 | SMPD3 | 0.0021 | 0.0209 | 0.0310 |
| 4 | 16 | cg09821790 | 68283575 | rs1122720 | 68412028 | NFATC3 | SMPD3 | 0.0008 | -0.0222 | 0.0229 |
| 4 | 16 | cg09821790 | 68283575 | rs8050499 | 68428326 | NFATC3 | SMPD3 | 0.0119 | 0.0229 | 0.0768 |
| 9 | 1 | cg20674738 | 26127400 | rs2786875 | 26061266 | PAQR7 | PAQR7 | 0.0001 | 0.0273 | 0.0069 |
| 9 | 1 | cg12709196 | 26289989 | rs2232648 | 26496455 | PAQR7 | CD52 | 0.0010 | 0.0097 | 0.0873 |
| 9 | 1 | cg12709196 | 26289989 | exm34246 | 26582091 | PAQR7 | CD52 | 0.0018 | 0.0082 | 0.0873 |
| 9 | 8 | cg23130097 | 119208244 | rs4876852 | 119542307 | NA | NA | 0.0008 | 0.0153 | 0.0834 |
| 10 | 1 | cg27170383 | 37692359 | rs3762352 | 38156902 | MEAF6 | SNIP1 | 0.0005 | 0.0092 | 0.0225 |
| 10 | 1 | cg27170383 | 37692359 | rs11264087 | 38163387 | MEAF6 | SNIP1 | <0.0001 | 0.0123 | 0.0021 |

Supp. Table 7. Association between top response SNPs for dominance effect and topic score. The associations between top SNPs and the response are indicated by the P-value (Res_pval). In cases where differential topics are associated with the SNPs (P-value < 0.1), P-value (Topic_pval) and correlation coefficient (Coef) are indicated. The chromosome (Chr) and genomic location (Pos) of each SNP are shown. The closest differentially expressed genes within 500kb are shown. Rows where SNPs are not associated with differential topics or have no proximal genes are denoted as NA.

| SNP | Res_pval | Topic | Topic_pval | Coef | Chrom | Pos | Gene | MAF |
| --- | --- | --- | --- | --- | --- | --- | --- | --- |
| rs6663620 | 1.82E-05 | NA | NA | NA | 1 | 90535344 | NA | 0.3423 |
| rs12746015 | 7.57E-05 | 5 | 0.0803 | 0.1709 | 1 | 212636824 | NA | 0.2477 |
| rs12746015 | 7.57E-05 | 9 | 0.0819 | -0.1708 | 1 | 212636824 | NA | 0.2477 |
| rs11130670 | 8.72E-05 | 4 | 0.0488 | 0.1953 | 3 | 58658454 | NA | 0.2928 |
| rs11130670 | 8.72E-05 | 9 | 0.0971 | -0.1649 | 3 | 58658454 | NA | 0.2928 |
| exm-rs7901695 | 4.71E-05 | 5 | 0.0717 | 0.1721 | 10 | 114754088 | TCF7L2 | 0.3018 |
| exm-rs4506565 | 4.71E-05 | 5 | 0.0717 | 0.1721 | 10 | 114756041 | TCF7L2 | 0.3018 |
| rs2090852 | 4.55E-05 | NA | NA | NA | 12 | 51086931 | ATF1 | 0.3378 |
| rs10783387 | 1.55E-05 | 5 | 0.0817 | 0.1673 | 12 | 51180143 | ATF1 | 0.3559 |
| exm1002721 | 2.39E-05 | NA | NA | NA | 12 | 51203371 | ATF1 | 0.3829 |
| exm2273492 | 8.50E-05 | NA | NA | NA | 12 | 51213765 | ATF1 | 0.3739 |
| rs10774961 | 3.59E-05 | 4 | 0.0058 | -0.2671 | 12 | 109518133 | ACACB | 0.4640 |
| rs12596798 | 7.68E-05 | 5 | 0.04 | 0.1972 | 16 | 8513277 | TMEM186 | 0.2928 |
| rs257868 | 8.29E-05 | 4 | 0.0698 | -0.175 | 16 | 29412503 | SPN | 0.4369 |
| psy_rs36025325 | 1.85E-05 | 10 | 0.097 | -0.1594 | 16 | 64075157 | NA | 0.3063 |
| rs6085122 | 4.85E-05 | 4 | 0.0584 | 0.1822 | 20 | 5329782 | PROKR2 | 0.2342 |
| rs6085122 | 4.85E-05 | 5 | 0.0108 | 0.2424 | 20 | 5329782 | PROKR2 | 0.2342 |
| rs6085122 | 4.85E-05 | 9 | 0.0386 | -0.1988 | 20 | 5329782 | PROKR2 | 0.2342 |
| rs6012442 | 9.25E-05 | 5 | 0.0157 | 0.2327 | 20 | 47105033 | ARFGEF2 | 0.2387 |


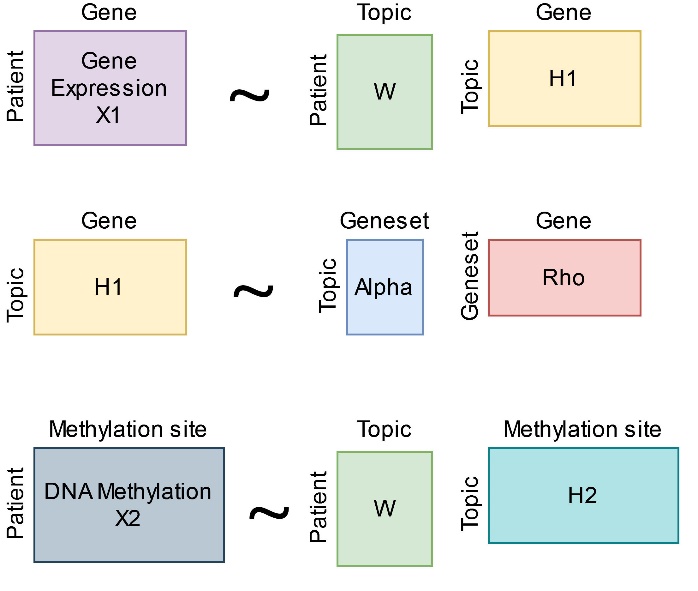


Supp. Figure. 1 Overview of the model design. Matrix decomposition in the model can be separated into the 3 steps illustrated in the figure.


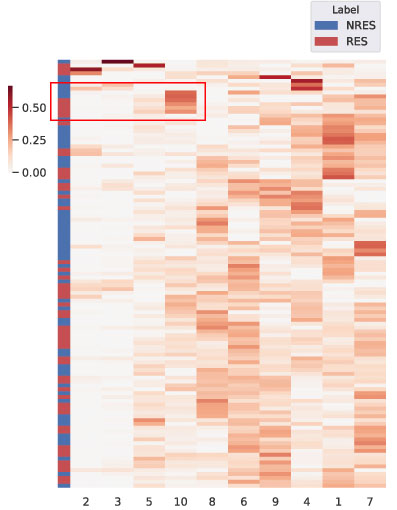


Supp. Figure 2. Hierarchical clustering of patient factor score. Each row represents a subject. The left portion indicates whether a patient is RES (red) or NRES (blue) based on the overall MADRS score. The right portion is the patient factor score. Each column represents a topic. The rows and columns are sorted by hierarchical clustering, with similar topics and subjects arranged closely. For differential topics, certain pattern of patient clustering can be observed. The red box indicates a patient cluster with higher topic 10 score.


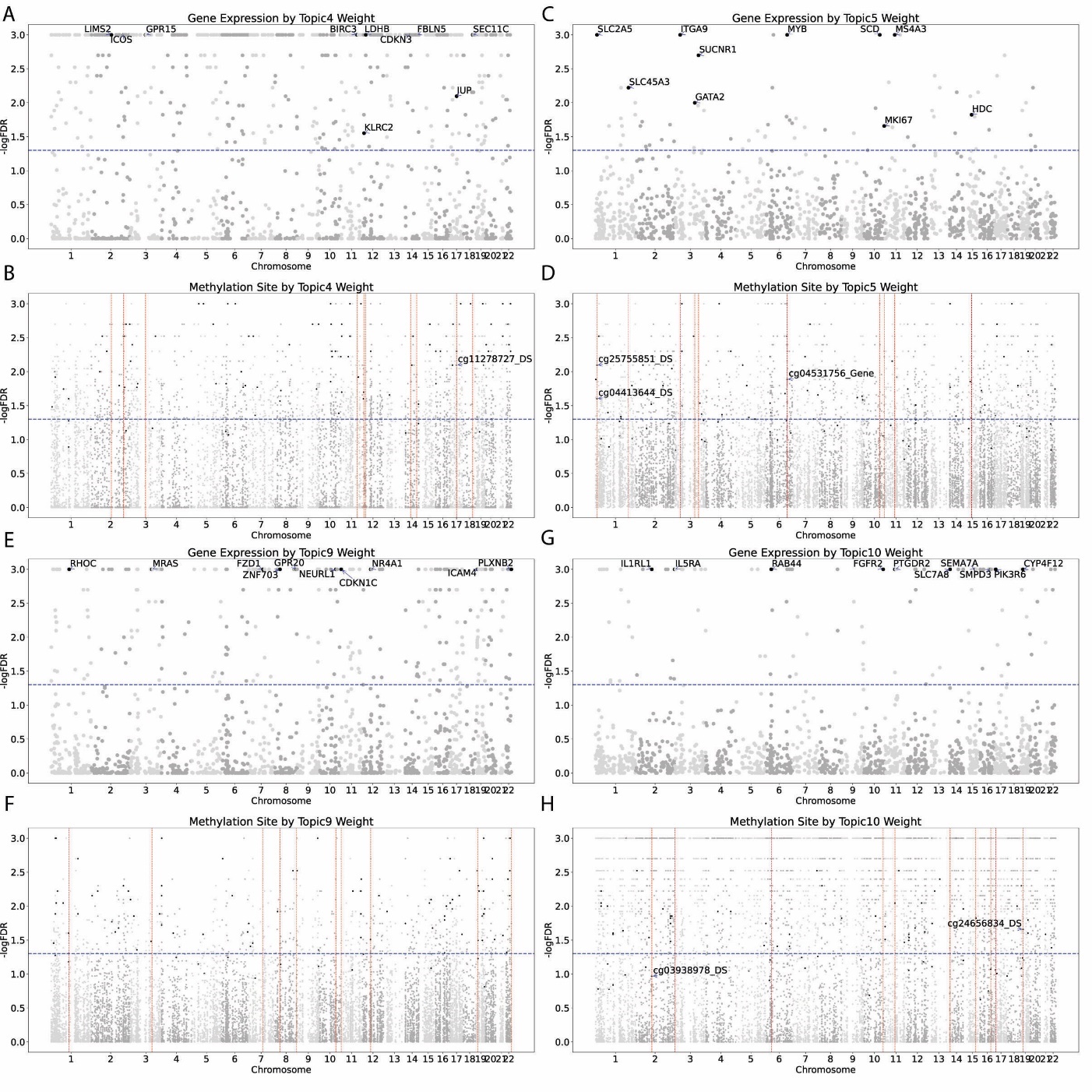


Supp. Figure 3. Gene and methylation Manhattan plots. (A) The Manhattan plot for gene expression of topic 4. (B) The Manhattan plot for methylation of topic 4. (C) The Manhattan plot for gene expression of topic 5. (D) The Manhattan plot for methylation of topic 5. (E) The Manhattan plot for gene expression of topic 9. (F) The Manhattan plot for methylation of topic 9. (G) The Manhattan plot for gene expression of topic 10. (H) The Manhattan plot for methylation of topic 10. Feature Manhattan plots are generated by permutation tests on feature factor scores with data shuffled over patients for 1000 models with fixed model factor score and identical hyperparameters based on the tested model. For each topic, the patient factor scores were tested against SNP variation by an OLS regression model, and the P-values were corrected by the BH procedure. Features are arranged by genomic location. Y axis shows the negative log P-value after permutation test. The blue horizontal line indicates the negative log P-value where P = 0.05. The red vertical line indicates the genomic location of top genes. In the top panel, the top 10 genes are highlighted in black with the name labeled. In the bottom panel, the top 100 methylation sites are highlighted in black. Among the top 100 sites, the ones proximal to the top genes have the name labeled with the relative position to the top genes (US, DS, and Gene for upstream, downstream, and within gene transcript respectively).


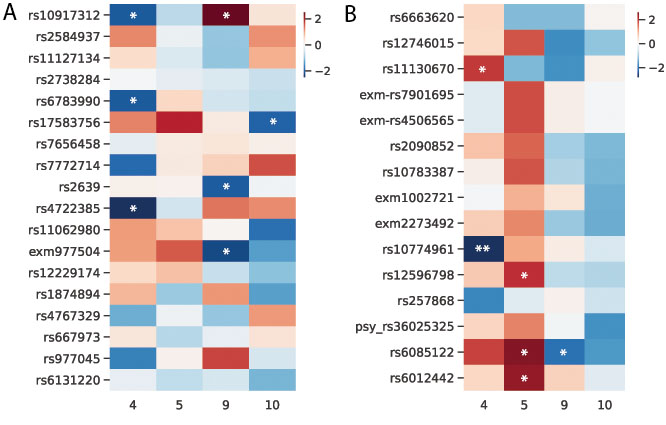


Supp. Figure 4. Heatmaps of t-statistics for correlation between top response SNPs and differential topics. (A) Heatmaps of t-statistics for correlation between top response SNPs and differential topics with minor allele frequency. (B) Heatmaps of t-statistics for correlation between top response SNPs and differential topics with alternative genotype grouping (B) as described in **Methods**. The asterisks * and ** indicate P-value < 0.05 and < 0.01 respectively.
